# A qualitative systematic review of experiences and perceptions of youth suicide

**DOI:** 10.1371/journal.pone.0217568

**Published:** 2019-06-12

**Authors:** Jessica Grimmond, Rachel Kornhaber, Denis Visentin, Michelle Cleary

**Affiliations:** 1 School of Nursing, College of Health and Medicine, University of Tasmania, Sydney, NSW, Australia; 2 School of Health Sciences, College of Health and Medicine, University of Tasmania, Sydney, NSW, Australia; Stellenbosch University, SOUTH AFRICA

## Abstract

**Background:**

Suicide remains a global issue with over 800,000 people dying from suicide every year. Youth suicide is especially serious due to the years of life lost when a young person takes their own life. Social interactions, perceived support, genetic predisposition and mental illnesses are factors associated with suicide ideation.

**Objectives:**

To review and synthesize qualitative studies that explored the experiences and perceptions of suicide in people 25 years old and younger.

**Design:**

Qualitative systematic review.

**Data sources:**

PubMed, PsycINFO, Scopus and CINAHL were searched alongside hand-searching reference lists up to October 2018.

**Methods:**

Methodological quality was assessed using the qualitative Critical Appraisal Skills Programme checklist. The 27 studies included in the review centered around youth suicide and included interviews with young people and members of the wider community. Thematic synthesis focused on factors leading to suicide attempts, elements important to recovery, beliefs within the community, and treatment/prevention strategies.

**Results:**

Thematic analysis of the articles revealed four categories: i) triggers and risks leading to suicidality; ii) factors involved in recovery; iii) need for institutional treatment/prevention strategies; and iv) beliefs about suicide at a community level. The first category was further subdivided into: i) behaviours; ii) feelings/emotions; iii) family influences; iv) peer influences; and v) other. The second category was split into: i) interpersonal; ii) cultural; and iii) individual influences, while the third category was divided into i) education; and ii) treatment.

**Conclusion:**

Youth suicide is a complex issue with many causes and risks factors which interact with one another. For successful treatment and prevention, procedural reform is needed, along with a shift in societal attitudes toward emotional expression and suicide.

## Introduction

Suicide impacts the lives of many people across the globe and is a concerning public health issue [1]. Almost 800,000 people’s deaths are the result of suicide internationally each year, accounting for 1.4% of all deaths [2]. The incidence of suicidal ideation universally increases during adolescence [3], with suicide the second leading cause of death worldwide in the 15 to 29 years age group [2]. Hence the impact of suicide on the families and communities is significant. Since the proportion of suicide deaths among young people is high, youth suicide should be considered a serious health issue due to the broader social cost and the years of life lost when a young person takes their own life.

A number of theoretical models provide a framework for understanding the complex interaction between biopsychosocial influences on suicidality. While each of these models provides a different explanation and emphasises different specifics, there are similarities throughout. The Interpersonal Theory of suicide (IT) [4], the Integrated Motivational-Volitional Model of suicidal behaviour (IMV) [5] and the Three-Step Theory (3ST) [6] each separate suicidal ideation from actual attempts and explore the differences between suicidal thoughts and suicidal actions. While the IT was developed to provide a comprehensible and potentially falsifiable framework of suicidality [4], the IMV model was borne out of a need to predict factors which influence suicide ideation and the circumstances whereby these thoughts are acted upon [5]. Similar to the IT, the 3ST is a demonstration of what Klonsky and May call an “ideation-to-action" framework [7] based in empirical evidence [6], though it emphasises different factors. Each of these models provides a detailed perspective of the cognitive, social and physiological contributors to suicidal ideation and attempts.

Psychological factors and personality differences such as hopelessness, impulsivity and resilience all have a bearing on a person’s likelihood of experiencing suicidal ideation [3]. Hence, identifying and understanding these factors is an important step in predicting and preventing suicide. The first step in the 3ST centres around experiences of hopelessness and pain which are usually, but not exclusively, emotional. [6]. They posit that frequent experiences of pain act as punishment, resulting in the individual “essentially being punished for living” [6], but that pain must also be coupled with psychological experiences of hopelessness [6]. Empirical data supports this, as both pain and hopelessness were strongly related to suicidal ideation and with one another [6]. While this theory is not specific to young people, the results remain consistent in the youth age bracket [6].

The IT also highlights the importance of psychological experiences. A key element of the IT is the perception of burdensomeness whereby low self-esteem and feelings of expendability (among others) contribute to dimensions of self-hate and liability [4]. These dimensions combine to result in perceived burdensomeness [4]. The IT suggests that when perceived burdensomeness is coupled with barriers to socialisation, and these are viewed as ‘stable and permanent’ [4] states, suicidal ideation may occur. These barriers to socialisation are termed ‘thwarted belongness’ and include feelings of loneliness and a lack of reciprocal care [4]. In this way, the IT provides a dynamic explanation of suicidal ideation as it considers the psychological influences in conjunction with social ones [4].

Other theoretical frameworks also consider social factors to have a strong influence on suicidality. In the second phase of the IMV model, the motivational phase, feelings of defeat and humiliation can progress to those of entrapment when threats to self-moderators, such as the inability to adequately resolve social problems, exist [5]. Like the IT, the IMV model considers thwarted belongingness to be an important social factor and, here, it acts as a motivational moderator which sees feelings of entrapment develop into suicidal ideation [5]. Research supports this model suggesting those who are more sensitive to the social judgements of others are more likely to feel defeat and entrapment, which are central to the motivational phase of the IMV model [5].

Socialisation is also considered a factor in the 3ST. The second step in this theory relates to connectedness which usually describes interpersonal relationships, but can also be extended to include an attachment to work or hobby interests [6]. In this model, connectedness protects against progression from moderate to severe ideation [6]. In this way, the 3ST differs from the IT, as it acknowledges factors which can stem progression [6], where the IT focuses on predictive factors alone [4]. In the 3ST, the psychological experiences of pain and hopelessness can lead to suicidal ideation, but only when these feelings are combined with a disruption to connectedness is it possible for a person to move from ideation to suicide attempt [6]. Hence, Klonsky and May [6] suggest that social influences play a vital role in suicide.

In line with these theoretical models, difficulty with socialisation and interpersonal conflicts have been identified as predictors of suicidality in adolescents [1]. The death of a loved one has been linked to suicidality in adolescents [1, 3] and research has identified that adolescents who have been exposed to suicide and related behaviours were far more likely to self-report similar related behaviours [8]. The causes for this link, however, are unclear. Experiencing the suicide of a family member may offer a behavioural model for young people who are already vulnerable [1, 5] or it may simply make it more salient in a young person’s mind as a solution to a problem [5].

In a similar way, the presence of ‘cluster effects’ and the concept of peer contagion highlights the important role of socialisation, especially in adolescents [9]. However, since the causes of these phenomena are poorly understood, misconceptions about the social transmission of suicide exist within society. The belief that incidents of suicide become higher when ideation and related behaviours are discussed is common and has informed public policy. These beliefs, however, are not based in empirical evidence [10]. Nonetheless, these ‘cluster effects’ do exist, and adolescence seems to be the peak time for peer contagion and social influence [9].

One explanation for these cluster effects is that people are more attracted to those who they perceive as being similar to themselves [11]. The group will likely experience similar stressors and events [1] and an adolescent’s experience of depression can be well predicted by those of their friends [9]. Therefore, rather than a ‘copycat’ explanation of suicide, it is reasonable to assume that some of the factors contributing to suicidal ideation in one person will also be affecting that person’s peers [9]. In fact, experiencing a peer’s suicide, itself, may elicit feelings of hopelessness and thwarted belongingness which are linked with suicidal ideation in the discussed theoretical models [4, 6].

The acceptance of suicide as an appropriate response to negative life events may also become normalised as a shared belief between members of social groups or certain subcultures [12, 13]. In this way, attitudes and understanding become shaped by the experiences and beliefs of others [12]. It is also important to consider, however, that those with pre-existing vulnerabilities may be drawn to likeminded people or subcultures (eg. Goth or EMO) from the outset, which will bias the sample and overemphasise the social influences on suicidality [12, 13].

 When considering the social impact that a family member’s suicide may have, it is impossible to separate the possible predisposition to psychiatric disorders associated with suicidality [1]. Autopsies indicate a prevalence of psychiatric disorders of over 90% in those who have died by suicide, though these are not always diagnosed while the person is alive [3]. A family history of mental health problems or suicide can also be a predictor of suicidality [4, 13], though the genetic influence is unclear.

These biological influences are also well explained in the current theoretical models. The IT explains that for a person to progress from ideation to attempt, they must acquire the capability for suicide [4]. This capability can be acquired cognitively, through a reduced fear of death, or physically through increased pain tolerance [4]. Similarly, the 3ST acknowledges that lower pain sensitivity can provide a dispositional capacity for suicide, while it can also be acquired through repeated exposure socially or through practical access to means [6]. It is clear through these models that physiology has a part to play in suicidality, but that both suicidal ideation and suicide attempts involve a complex combination of biopsychosocial factors.

Since there are so many influences on suicidality, identifying them and exploring their relationships to one another is an important step in prevention and treatment approaches. It is also important to consider that, while the current models of suicide provide a strong framework of the issue, they are not age specific. Since the experiences of adolescents will vary greatly from those further into adulthood, research that centres on young people is necessary [3]. While information gathered from quantitative studies is important in assessing various aspects of youth suicide, detailed thematic analysis of qualitative data [14] can provide unique and specific insights into the thoughts and feelings of those directly affected, as well as the wider community.

The need to explore societal perceptions of suicide has been identified [3] and the ability to compare these views with the lived experience of suicidal young people, for example, can provide a deeper understanding of the issue. Hawton, Saunders and O’Connor [1] have also suggested that future research should explore the factors that assist in moving away from suicidality. While various studies focus on specific aspects of suicide such as prevention strategies [15, 16], psychiatric factors [17], and treatment strategies, there is a need for a review which takes a more macro approach. Therefore, this review focuses on the suicidal behaviour of young people and explores not just the experiences of suicidal people, but includes the opinions of health professionals, parents and members of the wider community in order to explore the true complexity of the issue.

### Objective of the review

To review and synthesize qualitative studies that explored experiences and perceptions of suicide in people 25 years old and younger.

## Methods

This qualitative systematic review was guided by the thematic synthesis methodology of Thomas and Harden [14] with reporting meeting the Enhancing Transparency in Reporting the Synthesis of Qualitative research statement (ENTREQ) consisting of 21 reported items (S1 Table) [18].

### Inclusion and exclusion criteria

Included studies met the following criteria: i) original qualitative studies published in peer-reviewed journals in the English language with no date restriction; ii) participants were either adolescents or young adults (25 years of age or younger) who had attempted suicide, friends or family members of those who had attempted suicide or experienced suicidal ideations, professionals working with young people or members of the wider community; iii) qualitative interviews primarily discussing youth suicide and suicidal ideation in young people. Excluded studies were abstracts, editorials, conference proceedings, theses, and secondary research sources (e.g. reviews). Studies which were quantitative were excluded.

### Search strategy

A comprehensive literature search was conducted up until October 2018 without time limits (by RK, MC) using four electronic databases: PubMed, Scopus, Cumulative Index of Nursing and Allied Health Literature (CINAHL), and PsycINFO. Boolean connectors AND and OR were used to combine the following MeSH and search terms: adolescen*, teenager*, suicidal ideation, suicide, attempted suicide, trigger*, risk factors, perception* and qualitative research. As each database uses different indexed terms, the search strategy was adapted for differences in syntax and indexed/MeSH terms for each database (S2 Table).

Title and abstract screening of all papers identified by the search strategy was independently performed by authors MC, RK and JG with reference to the published inclusion/exclusion criteria.

### Search outcome

**Fig 1:**
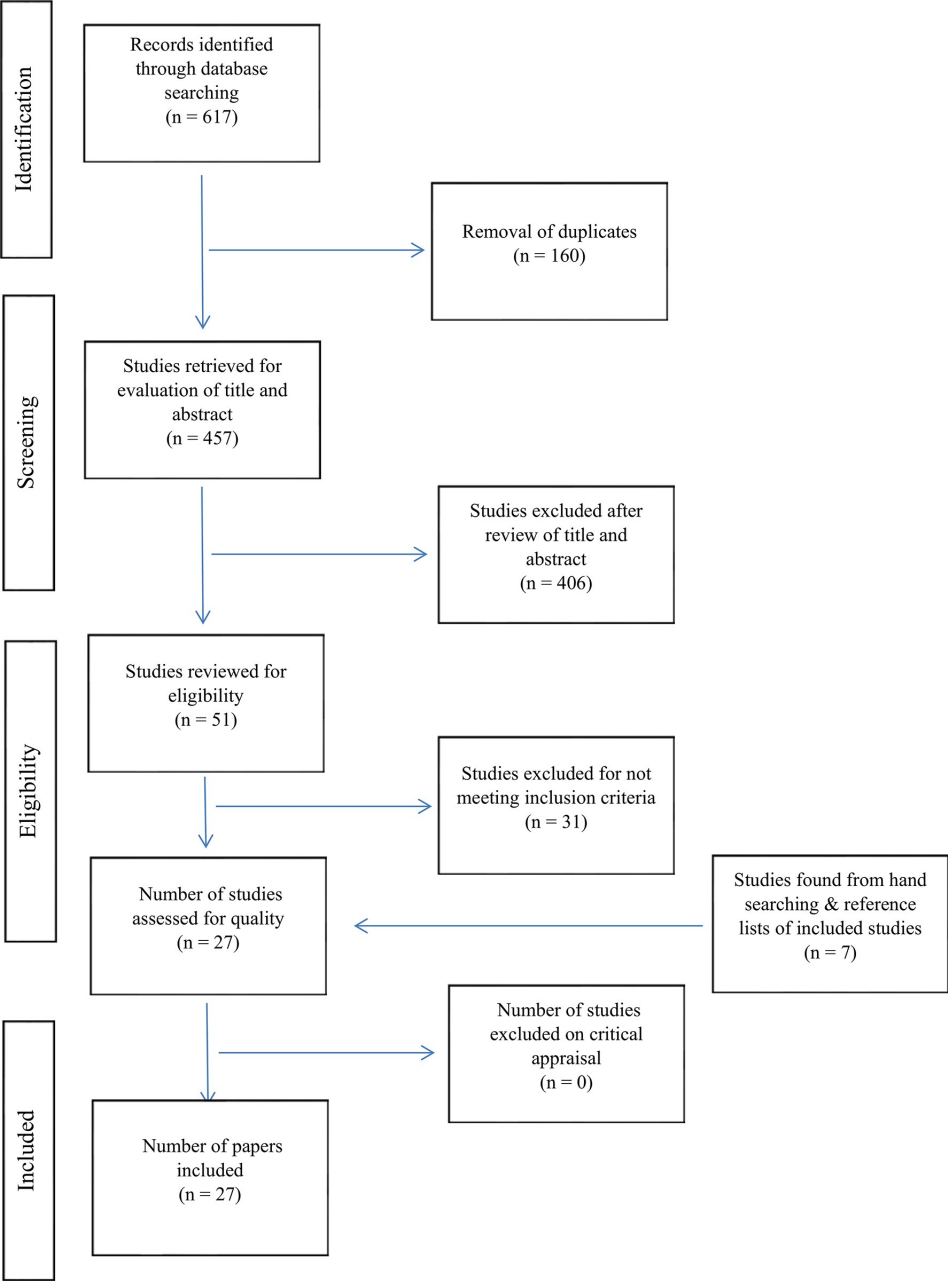
PRISMA flow chart search strategy.

A total of 617 studies were identified. Following removal of 160 duplicates, 457 title and abstracts were then screened of which 406 did not meet the inclusion criteria. Fifty-one full text articles were therefore retrieved and screened for eligibility and 31 were excluded. Review of the reference lists of the remaining studies identified 7 further studies meeting the inclusion criteria. In total, 27 qualitative studies met the inclusion criteria for this systematic review. The study selection process is detailed in the PRISMA Flow Diagram [19] (Fig 1).

### Quality appraisal

Quality appraisal of studies meeting the inclusion criteria was conducted by three members of the research team (MC, RK and JG) using the Critical Appraisal Skills Programme for Qualitative Studies Checklist [20]. All 27 qualitative studies identified were included (Tables 1–4).

**Table 1:** CASP ratings.

Authors and year of publication	Bergmans, Langley, Links, and Lavery (2009)			Bostik and Everall (2007)			Coggan, Patterson, and Fill (1997)			Everall, Bostik, and Paulson (2005)			Everall, Bostik, and Paulson (2006)			Fullagar, Gilchrist, and Sullivan (2007)			Gulbas, Hausmann-Stabile, De Luca, Tyler, and Zayas (2015)		
CASP Question	Yes	Can’t tell	No	Yes	Can’t tell	No	Yes	Can’t tell	No	Yes	Can’t tell	No	Yes	Can’t tell	No	Yes	Can’t tell	No	Yes	Can’t tell	No
Are the aims stated clearly?	●			●			●			●			●			●			●		
Is the qualitative methodology appropriate	●			●			●			●			●			●			●		
Is the research design appropriate to address aims of the research	●			●			●			●			●			●			●		
Recruitment strategy appropriate to aims?	●			●			●			●			●			●			●		
Does the data collection method addressed research issue?	●			●			●			●			●			●			●		
Has relationship between researcher and participants been considered?		●			●		●				●		●			●				●	
Have ethical issues taken into consideration?	●			●			●			●			●			●			●		
Was the data analysis sufficiently rigorous?	●			●			●			●			●			●			●		
Is there clear statement of findings?	●			●			●			●			●			●			●		
How valuable is the research?	●			●			●			●			●			●			●		

**Table 2:** CASP ratings.

Authors and year of publication	Gulbas and Zayas (2015)			Herrera, Dahlblom, Dahlgren, and Kullgren (2006)			Holliday and Vandermause (2015)			Jegannathan, Kullgren, and Dahlblom (2016)			Jo, An, and Sohn (2011)			Keyvanara and Haghshenas (2011)		
CASP Question	Yes	Can’t tell	No	Yes	Can’t tell	No	Yes	Can’t tell	No	Yes	Can’t tell	No	Yes	Can’t tell	No	Yes	Can’t tell	No
Are the aims stated clearly?	●			●			●			●			●			●		
Is the qualitative methodology appropriate	●			●			●			●			●			●		
Is the research design appropriate to address aims of the research	●			●			●			●			●			●		
Recruitment strategy appropriate to aims?	●			●			●			●			●			●		
Does the data collection method addressed research issue?	●			●			●			●			●			●		
Has relationship between researcher and participants been considered?		●		●			●			●				●		●		
Have ethical issues taken into consideration?	●			●			●			●			●				●	
Was the data analysis sufficiently rigorous?	●			●			●			●			●			●		
Is there clear statement of findings?	●			●			●			●			●			●		
How valuable is the research?	●			●			●			●			●			●		

**Table 3:** CASP ratings.

Authors and year of publication	Matel-Anderson and Bekhet (2016)			Montreuil, Butler, Stachura, and Gros (2015)			Orri et al. (2014)			Roen, Scourfield, and McDermott (2008)			Schwartz, Pyle, Dowd, and Sheehan (2010)			Shilubane, Ruiter, Bos, Reddy, and Van Den Borne (2014)			Shilubane et al. (2012)		
CASP Question	Yes	Can’t tell	No	Yes	Can’t tell	No	Yes	Can’t tell	No	Yes	Can’t tell	No	Yes	Can’t tell	No	Yes	Can’t tell	No	Yes	Can’t tell	No
Are the aims stated clearly?	●			●			●			●			●			●			●		
Is the qualitative methodology appropriate	●			●			●			●			●			●			●		
Is the research design appropriate to address aims of the research	●			●			●			●			●			●			●		
Recruitment strategy appropriate to aims?	●			●			●			●			●			●			●		
Does the data collection method addressed research issue?	●			●			●			●			●			●			●		
Has relationship between researcher and participants been considered?		●		●			●				●			●		●				●	
Have ethical issues taken into consideration?	●			●			●			●			●			●			●		
Was the data analysis sufficiently rigorous?	●			●			●			●			●			●			●		
Is there clear statement of findings?	●			●			●			●			●			●			●		
How valuable is the research?	●			●			●			●			●			●			●		

**Table 4:** CASP ratings.

Authors and year of publication	Strickland and Cooper (2011)			Strickland, Walsh, and Cooper (2006)			Sukhawaha, Arunpongpaisal & Rungreangkulkij (2016)			Tingey et al. (2014)			Walls, Hautala, and Hurley (2014)			White and Morris (2010)			Zayas, Gulbas, Fedoravicius, and Cabassa (2010)		
CASP Question	Yes	Can’t tell	No	Yes	Can’t tell	No	Yes	Can’t tell	No	Yes	Can’t tell	No	Yes	Can’t tell	No	Yes	Can’t tell	No	Yes	Can’t tell	No
Are the aims stated clearly?	●			●			●			●			●			●			●		
Is the qualitative methodology appropriate	●			●			●			●			●			●			●		
Is the research design appropriate to address aims of the research	●			●			●			●			●			●			●		
Recruitment strategy appropriate to aims?	●			●			●			●			●			●			●		
Does the data collection method addressed research issue?	●			●			●			●			●			●			●		
Has relationship between researcher and participants been considered?	●			●			●			●			●			●			●		
Have ethical issues taken into consideration?		●		●			●			●			●			●			●		
Was the data analysis sufficiently rigorous?	●			●			●			●			●			●			●		
Is there clear statement of findings?	●			●			●			●			●			●			●		
How valuable is the research?	●			●			●			●			●			●			●		

### Characteristics of studies

The studies included in this review ranged in size from a case study of one individual [21] to 134 participants [22] with an average of approximately 38 participants, though not all studies disclosed exact numbers. At least five studies involved Caucasian participants [21, 23–26], four included Native American populations [27–30] while four involved Hispanic and Latina participants [23, 31–33]. At least one study each focused on participants from Nicaragua [34], Korea [22], Iran [35], Italy [36] and Cambodia [37], while other studies referenced included participants from a variety of ethnic backgrounds [38, 39]. In all studies which provided the gender of participants, there were more female participants, except one which had equal representation of gender [36]. Five included studies reported only female participants [21, 31–34]. Participants who attempted suicide ranged in age from 11 to 28 years and had experienced suicidal ideation or attempted suicide at 25 years of age or younger. Studies which included data from other members of the community included participants aged up to 64 years old. Most studies involved data from young people who had experienced suicidal ideation and some additionally provided their parent/caregiver’s testimony [32, 40, 41]. Some studies focused on data from members of the wider community and their thoughts about youth suicide [23, 26, 28, 30, 37, 39, 42] or those who worked with [43] or knew [44] a young person who had attempted suicide. At least three of the included studies took place in schools [37, 44, 45], while others included interviews in treatment facilities [36, 40, 43] and post-discharge [34, 46]. Table 5 presents a summary of included studies.

**Table 5:** Summary of included studies (n = 27).

Author/s, year & country	Aims	Sample & study population	Methods	Findings
**Bergmans, et al. [47]**, 2009, Canada	To understand the transition to safer behaviours and to provide clinical suggestions to those who provide care to this population.	Sixteen people aged 18–25 years with a history of two or more suicide attempts.	Qualitative, grounded theory study	A pathway from high to lower risk was established. Young people at the highest risk of attempting suicide spoke about “Living to Die” and progressed through “Ambivalence and Turning Points” to “Pockets of Recovery” as they move aware away from suicidality.
**Bostik and Everall [24]**, 2007, Canada	To develop an understanding of adolescent’s perceptions of the role of attachment relationships in the process of overcoming suicidality.	Fifty adolescents who were previously suicidal between the ages of 13 and 19 years old.	Qualitative interviews, grounded theory study	Parents, peers and extra-familiar adults each had important attachment relationships with young people recovering from suicidal ideation as did a spiritual connection. Finding acceptance, having a permanent relationship, receiving encouragement and experiencing intimacy and closeness were all common experiences of attachment. These attachment relationships and experiences helped young people change their self-perceptions.
**Coggan, et al. [42]**, 1997, New Zealand	To enhance knowledge of ways to address youth suicide.	School age students	Focus groups and analysis	Students spoke about what they believed the warning signs of a suicidal friend, their perceptions of available services and resources for young people at risk and potential prevention strategies.
**Everall, et al. [21]**, 2005, Canada	To illustrate the role developmental processes, cognitive development, identity formation and autonomy seeking played in one teenager’s experience of becoming and overcoming being suicidal.	One 20-year-old female	Case study	The participant spoke about factors which contributed to her becoming suicidal and those that were important in overcoming suicidality.
**Everall, et al. [25]**, 2006 Canada	To explore how adolescents and emerging adults conceive their emotional experiences associated with being suicidal.	Forty-one females, nine males previously suicidal between the ages of 14–24	Qualitative interviews, grounded theory approach	Participants spoke of the overwhelming despair, shame and self-loathing, and alienation and isolation they experienced. They also spoke of how they responded to these emotions and how they moved beyond suicidality.
**Fullagar, et al. [26]**, 2007, Australia	To explore how everyday understandings of the issues surrounding suicide risk and prevention were constructed within community contexts and were mediate by a range of social institutions.	Eighty-one young people (aged 15–24 years), service providers (teachers, school counselors, sports coaches and youth and health workers) and adults (parents and community leaders)	Structured interviews including 10 open ended questions	Constructions of suicide through statistics and stories were common, as was discussion about stigma and distancing of suicide. Young people and adults also differed on their perspectives of youth suicide.
**Gulbas, et al. [31]**, 2015, USA	To describe and compare the conditions and experiences that precede the decisions to self-harm in order to contribute to an understanding of the contexts surrounding self-harmful behaviours within in Latina adolescent behaviour.	Thirty-seven Latinas between the ages 11–19 who attempted suicide through self-harm.	Qualitative interviews and analysis	Attempters spoke about feeling powerless, as well as unloved and unsupported in their interpersonal relationships. A history of self-harm was also common.
**Gulbas and Zayas [32]**, 2015, USA	To link the attempters experience to the broader socio-cultural forces that the attempters both encounters and surrenders to.	Ten Latina teen suicide attempters and their parents	Qualitative interviews and comparative analysis	Participants reported subjective distress, interpersonal discord and emotional isolation.
**Herrera, et al. [34]**, 2006, Nicaragua	To explore perceived causes and discover triggers and processes leading to suicidal behaviour among adolescent girls in Leon, Nicaragua.	Eight Nicaraguan girls aged between 12–19 admitted to hospital after attempting suicide	In-depth interviews, grounded theory and content analysis	Perceived causes were material conditions, family structure and norms and values. When combined with triggering events and emotions these conditions elicit action from the participants. These actions were explained as problem solving, escape or suicide attempt.
**Holliday and Vandermause [38]**, 2015, USA	To describe and interpret the phenomenon of attempted suicide in a sample of teens who visit an emergency department for a suicide attempt	Six young people aged 15–19 who visited an emergency department for a suicide attempt	Heideggerian hermeneutic methodological approach	Common patterns and themes were identified. The pattern of attempting as a way of communication was evident through themes of Ambiguity and cries of pain. The second pattern, attempting as transforming is described through being unconnected, spiraling down and being alone with suffering. Conversely, connecting was seen as a way to climb upwards.
**Jegannathan, et al. [37]**, 2016, Cambodia	To explore the views of the focus group on the societal attitudes towards suicide, contemporary media norms, Buddhism and their influence on suicidal behaviour	Forty-eight students from 2 schools in Cambodia	Focus groups and thematic analysis	The social stigma of suicide was a common theme throughout, as was the double-edged nature of the media as both educative and suicide-provocative. Suicide-ambiguity in Buddhism was also prevalent.
**Jo, et al. [22]**, 2011, Korea	To understand the suicidal ideation of the qualitative content analysis in South Korean college students	One hundred and thirty-four South Korean college students	Non-structured open questions, qualitative content analysis	Physical, physiological and social concerns were discussed as being facilitators of suicidal ideation, while religious, individual and relational beliefs were seen as inhibitors.
**Keyvanara and Haghshenas [35]**, 2011, Iran	To illuminate the socio-cultural context of attempted suicide among Iranian youth.	Twenty-five young people aged 14–17 who attempted suicide and were admitted to hospital in Isfahan	Qualitative interviews, thematic analysis	Participants frequently reported despair, failure in love, family issues involving conflicts between children and their parents and/or siblings, the pressure of high expectations from family and peers, and poverty as important factors in their suicide attempt.
**Matel-Anderson and Bekhet [43]**, 2016, USA	To explore components of resilience in adolescents who survived a suicide attempt from the perspective of nine psychiatric nurses.	Nine psychiatric nurses.	Focus group and analysis	Risk factors were split into six categories: Unstable households and traumatized childhood history, having a means to drugs and firearms, bullying, cognitive distortions and lack of vision for the future, absence of parental bonding and lack of positive role model, and poor self-esteem and issues with identity.
**Montreuil, et al. [40]**, 2015, Canada	To find what the perceptions of children with suicide associated risk factors and their parents are regarding helpful nursing care in pediatric mental health settings?	Children with at least one suicide risk factor and their psychiatric problem, and their parents	Semi-structured interviews, participant observation and inductive analysis	Caring for the child as a special person was considered important in recovery. Ways of doing this included getting to know the child, personalizing care, being available, and communicating calmly. Caring for parents was achieved through being available to parents and reassuring parents through talking. Managing the child’s illness involved including parents in the care team, linking the body to the thinking and teaching coping behaviours. It was also important to create a therapeutic environment by managing the physical and social environments.
**Orri, et al. [36]**, 2014, Italy	To explore the perspective of adolescents directly involved in suicidal acts	Sixteen adolescents with either single or multiple suicidal acts in their past or with a history of.	Qualitative interviews and interpretive phenomenological analysis	Common themes were divided into individual and relational dimensions of the suicide attempt. Individual dimensions included negative emotions toward the self and the need to have control over their lives. Relational dimensions involved a perceived impasse in interpersonal relationships, communication issues, and suicide as revenge.
**Roen, et al. [39]**, 2008, UK	To consider how some young people become positioned as suicidal subjects while others do not and how some young people find suicidal behaviour imaginable while others do not	Sixty-nine people aged 16–24 years	Interviews and focus groups, discourse analysis	Common themes for discussion involved the ‘othering’ of suicide, suicidal subjecthood as being readily accessible and attempts to rationalize why people attempt suicide.
**Schwartz, et al. [23]**, 2010, USA	To understand the attitudes, beliefs and perceptions of adolescents and parents of adolescents from a variety of backgrounds regarding adolescent suicide	Ninety-six children of 13–18 years of age and parents/guardians of children aged 13–18 years	Semi-structured focus groups, analysis	Participants spoke about the risk factors, predictability, preventability and environmental factors of suicide in young people,
**Shilubane, et al. [46]**, 2012, South Africa	To identify psycho-social target points for future educational interventions	Fourteen adolescents who recently attempted suicide	One-on-one in-depth interviews, analysis	Psychosocial factors identified in this study were disturbed family relationships and perceived accusations of negative behaviour. Problems with social support systems, such as family and peer problems, and experiences of negative emotions and depression were also prevalent. Participants discussed negative life events, often a family history of suicide, peer suicide or the individual’s previous suicide attempts, as well as the impact of living circumstances and a lack of knowledge of available counsellors.
**Shilubane, et al. [44]**, 2012, South Africa	To describe the impact on high school students of a suicide or suicide attempt by a peer to assess students’ knowledge about suicide, perceived risk factors, signs of poor mental health, and to assess their awareness of available mental health care and resources and opinions on prevention	Fifty-six high school student who had a peer commit or attempt suicide	Focus groups, inductive analysis	Peer reactions were discussed, as were the signs of poor mental health, the perceived cause of the peer’s suicide attempt/suicide, perceived availability of resources and opinions on prevention.
**Strickland and Cooper [27]**, 2011, USA	To gain an understanding of the moving processes and stories from the view of youth by focusing on the experiences of ‘at-risk’ Indian youth residing in a Pacific Northwest tribe.	Thirty ‘At-risk’ American Indian youth aged between 14 and 19 years old residing in a Pacific Northwest tribe.	Focus groups and observation, content analysis	Participants spoke of the effects that getting into trouble could have and ways of dealing with the trouble and coping with the stress. Staying on track was also considered an important focus for at-risk youth.
**Strickland, et al. [28]**, 2006, USA	To gain parents and elders perspectives on community needs and to identify strengths on which the community might build to reduce suicide	Forty-nine American Indian parents and elders	Focus groups, content analysis	Discussions centred around a loss of culture and tradition, and a breakdown of family values. Participants also considered that a connection to culture and community can protect against suicidality.
**Sukhawaha, et al. [41]**, 2016, Thailand	To understand and describe the triggering factors associated with suicidal attempts in adolescence from the perspective of adolescents who had direct experience with suicidal attempt by exploratory descriptive study.	Eighteen adolescents who had attempted suicide and some of their parents.	In-depth interviews, content analysis	Triggering factors included severe verbal criticism, unwanted pregnancies and mental illness causing intense emotions and irresistible impulses.
**Tingey, et al. [29]**, 2014, USA	To develop the Apache conceptual model of youth suicide with qualitative data from a community-based sample of Apache adolescents who have attempted suicide.	Twenty-two native adolescents who had attempted suicide	Longitudinal interviews, qualitative descriptive approach	Individual factors contributing to suicide involved emotion recognitions and dysregulation, and impulsivity and reactivity. Family factors included family dynamics, household composition, substance use and family support. Community factors involved grief burden, and stigma, while societal factors were imitation and minimizing the significance of youth suicide after the fact.
**Walls, et al. [30]**, 2014, Canada	To share the voices of adult community members from a single cultural group across 3 separate central Canadian first nations reserves who participated in focus group discussions about the devastating loss of their young people to suicide.	Elders and service providers	Qualitative interviews, thematic analysis	Participants spoke of interpersonal factors such as the presence of suicide clusters/normalization of suicidality, barriers to communication and relationships/early dating. Meso-level factors included family and community factors. Family factors involved the parental abuse of alcohol and drugs, gambling and poor parenting skills. Macro-level factors such as historical trauma, the effects of European contact and residential school systems, loss of identity and the need to return to the traditional way of life were all discussed in these focus groups.
**White and Morris [45]**, 2010, Canada	To document the planning and implantation of a four-part, classroom-based suicide prevention education program within one secondary school as a way to better understand how it is conceptualized and experienced.	Two grade 11 English classrooms scheduled to receive a suicide prevention curriculum	In-depth case study discursive critical constructionist methodology	Depression and mental illness, stress, uncertainty, multiplicity and unpredictability were all common perceptions of youth suicide. It was also observed that children often learned things which were not actively taught and the role this may play in planning education programs.
**Zayas, et al. [33]**, 2010, USA	To explore what the conditions in which suicide attempts occur among young Latinas, how Latinas experience the circumstances that led to the attempt and what young women say precipitated their suicide attempts and what triggers the act.	Twenty-seven teenage Latinas aged 11–19 living in New York City who had attempted suicide	Qualitative interviews, thematic analysis	Participants varied in why they attempted suicide and patterns of distress involving escalating tensions and a trigger were prevalent. These teenagers also spoke about their reactions, regrets and insights following their attempts.

### Data extraction and synthesis

Researcher JG in collaboration with other team members (MC, RK) led the data extraction and synthesis of these studies. Key themes were compiled for each article and these themes were then grouped based on common traits. In line with Thomas and Harden’s [14] methods for thematic synthesis, the ‘Results’ section of each article was analysed using line-by-line coding. Each category was designated a colour and the sub-categories within each theme were represented by a number. Text that was relevant was highlighted in the theme’s corresponding colour and the number of the subtheme written adjacent. Coloured tabs were used to mark the page to ensure each piece of relevant text could be quickly and appropriately accessed for further analysis. Upon completion of the line-by-line coding, each subtheme was re-examined, and each piece of text compared to others in that category for similarities and differences. The findings were then reviewed by all team members and synthesised.

### Results of the review

Thematic analysis of the articles revealed four categories: i) triggers and risks leading to suicidality; ii) factors involved in recovery; iii) need for institutional treatment/prevention strategies; iv) beliefs about suicide at a community level (Table 6). The first category, triggers and risks leading to suicidality was further subdivided into: i) behaviours; ii) feelings/emotions; iii) family influences; iv) peer influences; and v) other. The second category, factors involved in recovery was further divided into: i) interpersonal; ii) cultural; and iii) individual influences, while the need for institutional treatment/prevention strategies category is split into i) education; and ii) treatment subcategories.

**Table 6:** Summary of key findings.

Categories	Sub-Categories	Themes
Triggers and Risks Leading to Suicidality	Behaviours	Risk taking/Impulsivity
		Self-Harm
		Lack of Future Orientation
		Mood Swings
		Communication Difficulty
	Feelings/Emotions	Lack of Control/ Powerlessness
		Ambivalence Toward Death
		Isolation
		Anger/Aggression
		Self-Esteem Issues
		Appeal of Death
	Family Influences	Difficult Family Relationships
		Poor Living Conditions
		Family Violence
		Death of a Loved One
	Peer Influences	Difficult Peer Relationships
		Academic Challenges
		Failure in Love
	Other	Previous Experience with Suicide
		Cultural Challenges
		Drugs/Alcohol Use
Factors Involved in Recovery	Interpersonal	Formation/Improvement of Relationships
		Supportive Families
		Changing/Managing Environment
	Cultural	Building Cultural/Family Values
		Spirituality
		Community Factors
	Individual	Improved Self-Esteem
		Learning About Feelings and Life
		Finding Future Orientation
Need for Institutional Treatment/Prevention Strategies	Education	Education in Schools
		Youth-Specific Initiatives
		Information Initiatives
		Specialised Support/Education for Parents
	Treatment	Professional Help (e.g. Counselling, help lines, psychologists etc.)
		Treating the Whole Person
		Better Assessment
		An Holistic Approach to the mind/body
		Realistic Discharge Expectations
Beliefs About Suicide Within the Wider Community		Social Stigma
		Gender/Racial Differences
		Desire to rationalize ‘Why’
		It is Common
		Media Representations of suicide
		**Relationship with Mental Illness**

### Triggers and risks leading to suicidality

#### Behaviours

The most commonly displayed behaviour was difficulty with communication, specifically with communicating personal feelings [25, 38]. This was in part a result of participant age with many younger people lacking the “vocabulary to talk about feelings” [25], instead relying on clichés and metaphors which inadequately expressed their true emotions [25, 29]. For others, communication issues stemmed from emotional barriers due to poor relationships [32, 34] including mistrust of those around them [30]. When communication was attempted, disclosure to friends and family can be difficult for the suicidal person, who often receives little sympathy or tough love [32, 34, 36, 39, 42]. In addition, their confidant may be compromised in their ability to support in part due to issues arising from being sworn to secrecy [30, 42].

This inability to communicate their emotions led some participants to engage in self-harm in an attempt to release their feelings physically [21, 31, 33]. Indeed, the suicide attempt itself may be interpreted as an attempt at communication, which was often an explicitly stated objective [21, 36, 38, 47]. Self-harm was also reported to be a way of regaining control over an aspect of the participant’s life [31, 36], to punish themselves for perceived deficiencies [25] or to distract themselves from overwhelming negative emotions [41].

Many participants in these studies expressed a lack of future direction or focus as being associated with suicidal behaviour [25, 29, 35, 43] with those attempting suicide reporting that they did not know what their purpose in life was [29], that they had lost their future [35, 43], and that they lacked goals and plans [25]. Other behaviours commonly reported were exhibiting mood swings [34, 42] and engaging in high-risk activities [25], often involving cars [23, 42].

#### Feelings/Emotions

The most frequently reported feelings associated with suicidality were those of worthlessness [31, 41, 47], self-loathing [25] and other general self-esteem issues [23, 43]. These issues sometimes stemmed from a dissatisfaction with one’s physical appearance [21, 22, 36] and are closely linked to the feelings of shame [21, 24]. These feelings were usually associated with rejection, either real or imagined, by family members [21, 25, 33] or peers [25, 33].

Physical and emotional isolation was a major contributing factor in suicidal behaviours. Participants felt that those around them would not understand their experiences [21, 32, 38, 46], that they were in some way different [25] or that no one cared [24]. Others feared the stigma associated with disclosing negative emotions [25]. The perception of isolation resulted in the person keeping their feelings a secret [34, 42, 45] and attempting suicide or self-harm was an alternative to expressing emotions [21, 33].

Feelings of anger and aggression were also prevalent in many studies [21, 35, 46]. The suicide attempt was a way of releasing the escalating anger within a young person [33, 35, 41]. This anger, described as “uncontrollable” and “a loss of self-control” [35], rarely had an obvious cause. Anger was described as the one emotion that “became a safe way to show the world some of the negativity that dominated their lives” [25]. The anger for some participants was so strong that their suicide attempt was intended as revenge designed to elicit guilt and despair from those around them [36].

The intense emotional and physical experiences led young people to form a particular opinion of death. To some, the negativity in their life resulted in death becoming an appealing concept as an “avenue to free them of the pain” [38], as “salvational” [36] and as “a comfort” [47]. In contrast, some reported a degree of ambivalence, not wanting necessarily to die, but not wanting to live [47], seeing no other alternative [34, 38] or simply being unable to specify their intent [33].

#### Family influences

Negative life experiences and triggering events, including the death of a family member or close friend [29, 34, 43, 46] played an important role in a young person’s decision to end their life. Difficult relationships with family members was the most commonly reported issue in the lives of suicidal youth. Major family issues identified were relationship struggles with their mothers [21, 28, 29, 33, 34, 41, 46], distant or unavailable fathers [21, 27, 34, 35, 46] and fractured families [23, 31, 35, 43]. Sibling conflicts also contributed to difficult family life [21, 27, 29, 35, 41, 46]. Harsh criticism and strictness from family members [21, 31–34, 41] and difficulty with communication within the family [25, 32, 35] were key factors contributing to a problematic family dynamic. Some believed their suicide attempt would improve their relationships with their families [31], though this did not always the eventuate [29]. In several studies, a history of family violence and childhood maltreatment was present [41, 43, 47] contributing directly to suicidal ideation [31–35].

#### Peer influences

While difficulties at home were the most important contributor, many studies also highlighted the importance of a young person’s social and school life. The academic challenges of schooling and the real or perceived underperformance at school resulted in low self- esteem [28, 31, 32], parental disappointment [31, 32, 35, 44], stress [31, 44] and bullying from peers [35]. In addition to the academic challenges, complex peer relationships were significant stressors. The importance of choosing the right friends [24, 27] who can be trusted [46], difficulty connecting with peers [22, 25, 36] and bullying arising from social issues [23, 28, 31, 33, 43, 44] were also common peer influences contributing to suicidality.

Romantic relationships are important for young people, and solace in these relationships was especially important for those struggling with family and peer relationships. The failure of these relationships or experiences of unrequited love were common sources of stress leading to suicidal ideation [23, 34–36, 43]. The failure of these relationships was sometimes due to family expectations [35]. The issue of teen pregnancy provides an emotional upheaval, in particular when it results in the breakdown of the relationship [30, 41, 44].

There were significant challenges faced by members of minority cultures. Experiences of prejudice [27, 31], the loss of one’s cultural identity [28, 30] and a longing to return to the country of one’s origin [31] were all identified as contributing to suicidal ideation. Differences in cultural identity also existed between parents and their children leading to conflict and differences in generational expectations regarding culture [32, 34].

#### Other

Involvement in a deviant peer group could lead to experimentation with illicit drugs and/or alcohol [21, 32]. Substance abuse, either by the young person [21, 24, 25, 27, 41, 42] or by a family member [23, 29, 30], was a common theme throughout the studies. For many attempting suicide, drugs and alcohol were believed to be taken as a way to escape from or cope with intense emotions [23, 25, 27, 42]. Drug ingestion was a common method of self-harm and suicide [23, 30, 43], while addiction was highlighted by the community as being associated with suicide [39, 42].

Other experiences which influenced suicidality included previous experience with suicide and poor living conditions. Repeat suicide attempts were present [46], while many had a family member or friend who had committed suicide, prompting discussion about copycat behaviour [29]. While the presence of suicide in a young person’s life is a risk factor and actual incidences of copycat behaviour were reported [29, 30] there was a broader perception of suicide clusters [26, 46] which is likely to overstate this phenomenon. Unideal living conditions and experiences of poverty were also discussed as an influence on youth suicide [30, 34, 35] with one participant stating that she attempted suicide because she wished to “ease the financial burden on family” [35].

### Factors involved in recovery

#### Interpersonal

Following suicidal ideation, a number of factors were identified as helpful in recovery. Most importantly, the development or improvement of interpersonal relationships with family members [21, 24, 29, 33, 36, 38], peers [24] and mental health professionals [38] can be particularly helpful in moving past suicidality. Connection with at least one other person [24, 33, 39, 43], including other patients in rehabilitation programs [47] was considered most important for successful recovery. This development of new relationships is essential in reconstructing their self-esteem [21, 24, 25], which is another key theme in recovering from suicidality.

Changing or managing the physical and social environment in which a young person lives, also assisted in the development of these stronger relationships and improved outlook on life. This may involve either relocating or changing schools [25]. Similarly, the development of stronger relationships with members of one’s family and community can help strengthen a person’s cultural and family value, [30, 39] and highlights the importance of a supportive family in recovery [42]. For some, the attempt itself prompted families to provide more support than they previously had [24] and this change in home environment assisted recovery. Managing the therapeutic environment by allowing children to bring their own bed covers to hospital and ensuring their room is physically comfortable (appropriate lighting and temperature) was considered supportive for children recovering from suicide attempts [40].

#### Cultural

Spirituality was another common theme throughout these studies. Some found that developing a stronger relationship with God [24] helped in their recovery, while others felt that their religion discouraged suicidality [22, 34, 37, 43]. Spirituality was not, however, always discussed as a way of recovering. Some felt that the tightness of their community’s church group would stop them disclosing suicidal feelings [42] and one participant believed their peer’s suicide was the result of a curse [44].

The importance of the wider community for recovery and prevention was a common theme. It was felt that young people were excluded or let down by the unwillingness of the community to adapt and include young people [26, 34]. In contrast, some Native American communities invited Elders to engage with younger members of their tribe which was helpful in bridging the generational gap [28, 30].

#### Individual

Some common themes related to changes in the individual’s mindset. Personal factors which were considered important in recovery were the development of future goals and direction, [25, 34, 43] and learning about life [39, 47] and feelings [43, 47].

### Need for institutional treatment/prevention strategies

#### Education

A number of common themes arose highlighting the need for education and who should receive it. Many participants emphasised the need for improved and more accessible information concerning youth suicide [40, 44, 46]. The need for education in schools regarding warning signs, access to help and risk factors were identified [23, 26, 42]. However, the possibility of negative consequences from education initiatives, such as the normalisation or encouragement of copycat behaviour was a concern [45].

#### Treatment

There were also several themes which explore the best methods for treatment and assistance with recovery. The ability to access the help of a health care professional was a reoccurring theme [22–24, 38, 46, 47], although it was not always identified as being useful [30, 42]. Youth-specific initiatives were widely considered beneficial [28, 42, 44], as was specialised support for parents [26, 30, 40, 43]. Other themes identified as useful in treatment involved treating the persons’ situation holistically [40], including better and broader assessment [38, 42, 43], more realistic discharge expectations [30, 43] and an holistic approach to the treatment of the mind and body [40].

### Beliefs about suicide within the wider community

At the community level, many studies explored commonly held beliefs within society. Most prevalent was the negative stigma associated with suicide [37, 39, 43]. Many attempting suicide believed they would be judged poorly following an attempt [25, 29, 33, 39] or for expressing negative thoughts and feelings [25, 26, 42]. A more subtle example of this stigma in society was the reoccurring ‘othering’ of suicide attempters by members of the community [26, 39]. In this process, people attribute a person’s suicidality to various demographic markers that are different to their own, such as age [39], socio-economic status, gender [26] or race [23]. This stereotyping removes themselves from alignment with the issue. Much of this ‘othering’ results from a genuine desire to understand why suicide occurs, however misplaced [39].

The association between suicide and mental illness was raised in many studies [25, 41, 45, 47], with issues relating to stigma highlighted. Media representations of youth suicide also influenced people’s perceptions [26, 37, 42], which may overemphasise the incidence [26].

## Discussion

The aim of this study was to review and synthesize qualitative studies that explored the experiences and perceptions of suicide in people 25 years old and younger. By including the experiences of suicidal young people alongside those of their friends and family, as well as the discussions with health professionals and members of the wider community, we hoped to find commonalities and differences which explore the true complexity of the issue. Findings were split into four broad themes which covered the risks and triggers for suicidal ideation, important protective factors in moving past suicidal ideation, areas in which treatment/prevention strategies could be improved and the beliefs held at a societal level. Due to the scope of our review, these four categories remain broad in order to capture the variety within them, and sub-categories and themes describe in more detail the specifics.

### Relationships between themes

Whilst triggers were assigned into one of five sub-categories (behaviours, feelings/emotions, family and peer influences and others), these terms are heterogenous and there is a high degree of interconnectedness between them. The negative social influences in a person’s life often lead them to exhibit behaviours or elicit feelings associated with suicidality. Similarly, protective factors often involve the resolution of previously triggering experiences or behaviours. It was also clear that some of the societal attitudes, such as the negative stigma of youth suicide and self-harm, can also be contributing factors in youth suicide. Evidently. while the subcategories have been reported separately, the inter-relationship between many of them is complex.

Participants frequently reported a lack of control over their lives [21, 31, 36], which often resulted in self-harm or the desire to end their lives [31, 36]. The lack of control was closely related to the absence of future orientation and goal exhibited by many participants [25, 29, 35, 36, 43]. The perceived or real inability of control their own lives resulted in many of the affected young people not demonstrating an interest in their future as they perceive that they cannot influence the outcome or plan for future choices including success or failure. In one instance, this powerlessness was the result of parental restrictions which resulted in the breakdown of the young person’s significant relationship [35]. The traumatic experience of a failed relationship was commonly the result of a difficult parental affiliation and caused the emotional experience of powerlessness in this important life event and hence future relationships.

Peer relationships also demonstrated a complex interplay between young peoples’ experiences and emotions. Performing poorly at school impacted a child’s relationship with their parents [31, 32, 35, 44] as well as providing a source of antagonism from peers at school [35]. The resulting perception of failure can increase feelings of isolation which has a significant negative impact on self-esteem for young people [31, 32]. The experience of being bullied was often cited as a source of stress. While sometimes the result of poor academic performance, cultural differences and prejudice were reported as important influences related to bullying [27, 31]. Negative associations with one’s culture as a result of this bullying can increase feelings of isolation and lead to the degradation of cultural value, which provide important protective factors for mental health issues [30, 39].

The cultural challenges a child faces may also stem from intergenerational differences. Parents who have migrated to another country may have stronger or different cultural values from those held by their children [32, 34]. Conflict can arise as the parent tries to enforce restrictions upon the child, resulting in resentment, animosity and, ultimately, a difficult parent-child relationship. These cultural differences between caregivers and children extend beyond the experience of migration. Many of the young people in the included studies have experienced fractured family structures [23, 31, 35] with some raised largely by grandparents [28, 43]. This generational difference and the shifting societal values over time can cause friction at home and add to already complicated relationships. It is also important to consider that, while difficult family relationships were a common source of stress leading to suicidality in these studies, the improvement of parent-child dynamics was also discussed as assisting in the recovery after attempted suicide [24].

### Relationships with empirical evidence

A number of the themes which emerged in this review are reflected in the current body of empirical evidence that exists on suicide and associated risk factors. Family influences, such as family violence [1, 3, 4] or the death of a loved one [1, 3] have been shown to be linked with suicidality, as have other social influences such as previous experience with suicide [4], experiences of bullying [1] and social isolation [4]. Individual factors such as impulsivity [4] and a lack of future direction [3], as well as a history of mental illness [4, 13], substance use [1, 13] or self-harm [13] can also be predictive of suicidal behaviour and were all themes of this review. Teaching school aged children skills in psychological assessment has had positive results in the reduction of suicidality [1], and the identification of the need for better education in schools is, therefore, supported by empirical evidence.

### Relationships with theoretical models

Many of the common themes found in this review also support the current theoretical models of suicide. One of the key components of the Interpersonal Theory of Suicide (IT) is the notion of ‘Thwarted Belongingness’ [4]. Themes such as isolation, difficult family and peer relationships and failure in love were common triggers/risks leading to suicidality and reflect a disconnect in belonging and connectedness, which are key components in both the IT [4] and the 3 Step Theory (3ST) [6]. The 3ST, however, explains that connectedness can also protect against suicidality [6] and this does appear consistent with the findings of this review. The formation/improvement of relationships, a spiritual connection and community factors were identified as common themes in recovering from suicidality.

Another key component of the IT is ‘Perceived Burdensomeness’ [4]. While none of the identified themes specifically reflected perceived burdensomeness, there were several examples of this reported in the included studies. One young person reported feeling that their family was suffering as a result of them [22], while a participant in another study suggested that taking her life would ease the financial burden on her family, who were living in poverty [35]. In one study, the father of a suicidal girl blamed her for having to cancel a trip to see extended family and costing him “thousands and thousands and thousands of dollars” [32]. While this is not the perception of the suicidal young person, it is reasonable to assume that the blame placed on her would result in the perception that she was a burden on her family. Though these examples were described by difficult family relationships and poor living condition themes, they are clear examples of how perceived burdensomeness was present throughout this review.

The final element in the IT is that one must acquire the capability for suicide [4]. Self-harm and previous experience with suicide are two ways that have been identified as providing the capability for suicide in the IT [4] and were commonly reported themes throughout this review. These themes are also Volitional Motivators in the Integrated Motivational-Volitional Model which contribute in the transition from suicidal ideation to attempts [5]. The use of drugs and alcohol was also a common risk/trigger for suicidality in young people and have been shown to decrease sensitivity to pain. Hence, the capability for suicide, as outlined in the IT [4], could also be acquired through substance use as a tolerance for pain is heightened.

The use of drugs/alcohol was identified as a common theme in this review. However, since this review includes such a wide range of participants, it is important to acknowledge the clear delineation between the beliefs held by those in the community about suicide and the discussions about actual instances of suicidal attempts. The use of drugs and alcohol was a common theme reported by those who attempted suicide [21, 25, 27, 41] including the direct influence of substance use by their family members [23, 29, 30]. However, some of the studies were based around the beliefs of those within the wider community [39, 42]. In these instances, discussion about substance use was also prevalent, but usually this involved the ‘othering’ of suicide attempters because of their association with drugs [39, 42] and the opinion that those who abuse drugs are more likely to commit suicide.

Other themes in this review also support various aspects of the current theoretical models. The 3ST explains how experiences of pain are important in developing suicidal ideation [6]. Experience with self-harm, family violence or poor living conditions may all be examples of physical pain experiences, while failure in love and difficult family and peer relationships were themes which reflect the emotional pain which may lead to suicidality. Similarly, communication difficulties and a lack of future orientation reflect Threats to Self-Moderators and Motivational Moderators, respectively, which are key elements of the IMV model which may lead to suicidality [5].

The death of a loved one and previous experience with suicide were common themes in this review. While these have been identified as ways of acquiring the capability for suicide and Volitional Motivators in the IT [4] and IMV Model [5], respectively, they also relate to theories of peer contagion [9] and the social transmission of suicidal ideation. Copycat behaviour was highlighted as a factor by some of the studies [29, 30], although the level of understanding around the mechanisms through which this occurs was low. While the suggestion that enquiring about suicidality can increase suicidal behaviour has been disproven [10], there are a number of other explanations for the presence of these ‘cluster effects’. It is possible that stressors affecting one member of a social group will also affect their peers [1], or that suicidal behaviour is normalized within the group [12, 13]. When the young person has experienced the suicide of a family member, especially a parent, there may also be genetic influences or behavioural modelling consequences present [1]. The themes that have emerged in this review which relate to death are, therefore, expected.

### Limitations

One limitation of this study was the broadness of scope. Including a significant number of studies has been useful in the ability of this review to address the range of topics of discussion and provides an excellent exploration of how young people’s experiences may differ from the beliefs and experiences of others. The studies ranged greatly in scope and participants, including interviews with attempters [24, 47], discussions with members of the community [26, 42] and interviews with health practitioners [43, 45]. The diversity of these studies allows for the emergence of themes which are common to a variety of situations and demonstrates the perceptions of triggers of youth suicide for those affected. However, the wide range of participants inhibits the detailed exploration and reinforcement of sub-categories. The in-depth reporting of relationships between themes and emergence of reoccurring, smaller themes is beyond the scope of this review and should be considered in future studies. Similarly, the single thematic output used to report the findings of this review does not allow for the detailed exploration of cultural differences between the included studies and the impact that religion and culture may have on suicidality in young people.

Another aspect of this review worth noting is that, while common stressors and risk factors have been identified through this review, they are not, in themselves, causes of suicidality. While difficult relationships with family members have been identified as the most common negative experience of those who attempted suicide, they are also common experiences for most young people which do generally not result in suicide attempt. It is important to consider that, while the triggers/risk factors identified in this review may be indicative of suicidal behaviour, they are not necessarily causes or even likely predictors. The experiences, both emotional and physical, that contribute to youth suicide can be exacerbated by mental illness and are unique to the individual and their circumstances. Mental illness is also under-diagnosed, particularly in young people, and hence this is not always identified before or even after a suicide attempt.

### Implications for future research and practice

One of the reoccurring sub-categories in this review was the perceived need for better education about youth suicide, especially in schools [23, 26, 42, 44] to assist with detection of warning signs and reduce the social stigma surrounding the issue. However, there was also concern about the possibility that youth suicide education could encourage copycat behaviour from other young people although this is not consistent with empirical research [10]. Dishion and Tipsord’s [9] study on peer contagion found that deviant behaviour appears to be more contagious during unstructured portions of interventions. Hence, it may be proposed that the negative consequences of peer contagion may be minimized by structured interventions and close supervision [9] and future research should explore the degree to which these programs have tangible benefits, rather than just filling a perceived need, and best practice in such programs.

The prevalence and consequences of the social stigma associated with suicidal ideation and emotional expression was a common theme of this review. As a result, it is clear that there is a need to reduce the stigma surrounding negative emotions and suicide ideation. Feelings of isolation result from an inability to communicate with others about feelings, often because the young person fears judgement [25, 26, 42] or believes they are alone in these feelings [21, 32, 38, 46]. Isolation can lead some young people to engage in self-harm and suicidal behaviour in an attempt to cope with or escape from the overwhelming emotions they feel they cannot share with others without being persecuted [21, 33]. This demonstrates a clear need to encourage communication and break down negative stigma within the wider community. Future research should explore the various aspects of this social stigma with an aim of identifying methods of reducing it and the effectiveness of program designs assessed through empirical research.

Another key theme of this review centred around the current treatment procedures following a suicide attempt and the perceived need for their improvement. Inadequate treatment for, and assessment of, young people experiencing suicidal ideation provide little or no improvement of the person often resulting in further suicide attempts. It is, therefore, important that these treatment and assessment practices are enhanced to be more effective and reduce incidences of youth suicide. This review highlights a number of ways that young people and practitioners perceive this could be achieved. Treating the individual as a whole person and considering their mental health, as well as their physical health, upon presentation post attempt was a widely reported area for improvement [40, 43]. Similarly, managing discharge expectations [43] and both treating and educating parents was also identified [26, 30, 40, 43]. Future research should explore the efficacy and need for these improvements to inform best practice in patient care post suicide attempt.

## Conclusion

The use of qualitative studies provides important insight into the lived experience of suicidal ideation in young people, and the experiences of their parents and practitioners. An understanding of the interplay of motivators of young people towards suicide requires the collection and synthesis of qualitative data which is similarly complex allowing for the full exploration of the various contributing factors. The diversity of the studies in this review allowed for the identification of the richness of factors in a young person’s life which contribute to suicidal ideation. Themes relating to a person’s recovery following an attempt, institutional treatment and prevention strategies and the beliefs held about suicide at the community level were also identified. The overlap between themes within and between these categories highlights the intricate nature of youth suicide which can inform future primary research studies, policy and clinical practice.

## Supporting information

S1 Table ENTREQ. (DOCX)

S2 Table Sample search strategy. (DOCX)

## References

[1] Hawton K, Saunders K, O'Connor RC. Self-harm and suicide in adolescents. Lancet. 2012;379(9834):2373–82. 10.1016/S0140-6736(12)60322-5 22726518

[2] World Health Organization. Mental Health, Suicide data, 2019. Available from: https://www.who.int/mental_health/prevention/suicide/suicideprevent/en/.

[3] O'Connor RC, Nock MK. The psychology of suicidal behaviour. Lancet Psychiatry. 2014;1(1):73–85. 10.1016/S2215-0366(14)70222-6 26360404

[4] Van Orden KA, Witte TK, Cukrowicz KC, Braithwaite SR, Selby EA, Joiner TE Jr. The interpersonal theory of suicide. Psychol Rev. 2010;117(2):575–600. 10.1037/a0018697 20438238 PMC3130348

[5] O'Connor RC, Kirtley OJ. The integrated motivational-volitional model of suicidal behaviour. Philos Trans R Soc Lond B Biol Sci. 2018;373(1754). 10.1098/rstb.2017.0268 30012735 PMC6053985

[6] Klonsky ED, May AM. The three-step theory (3ST): a new theory of suicide rooted in the “ideation-to-action” framework. Int J Cogn Ther. 2015;8(2):114–29. 10.1521/ijct.2015.8.2.114

[7] Klonsky ED, May AM. Differentiating suicide attempters from suicide ideators: a critical frontier for suicidology research. Suicide Life Threat Behav. 2014;44(1):1–5. 10.1111/sltb.12068 24313594

[8] McMahon EM, Corcoran P, Keeley H, Perry IJ and Arensman E. Adolescents exposed to suicidal behavior of others: Prevalence of self‐harm and associated psychological, lifestyle, and life event factors. Suicide Life Threat Behav. 2013; 43(6): 634–45. 10.1111/sltb.12045 23855284

[9] Dishion TJ and Tipsord JM. Peer contagion in child and adolescent social and emotional development. Annu Rev Psychol. 2011; 62: 189–214. 10.1146/annurev.psych.093008.100412 19575606 PMC3523739

[10] Dazzi T, Gribble R, Wessely S and Fear NT. Does asking about suicide and related behaviours induce suicidal ideation? What is the evidence? Psychol Med. 2014; 44(16): 3361–3. 10.1017/S0033291714001299 24998511

[11] Cemalcilar Z, Baruh L, Kezer M, Kamiloglu RG and Nigdeli B. Role of personality traits in first impressions: An investigation of actual and perceived personality similarity effects on interpersonal attraction across communication modalities. J Res Pers. 2018; 76: 139–49. 10.1016/j.jrp.2018.07.009

[12] Trnka R, Kuska M, Balcar K and Tavel P. Understanding death, suicide and self-injury among adherents of the emo youth subculture: A qualitative study. Death Stud. 2018; 42(6): 337–45. 10.1080/07481187.2017.1340066 28590823

[13] Young R, Sweeting H and West P. Prevalence of deliberate self harm and attempted suicide within contemporary Goth youth subculture: longitudinal cohort study. BMJ. 2006; 332(7549): 1058–61. 10.1136/bmj.38790.495544.7C 16613936 PMC1458563

[14] Thomas J and Harden A. Methods for the thematic synthesis of qualitative research in systematic reviews. BMC Med Res Methodol. 2008; 8:(1) 45 10.1186/1471-2288-8-45 18616818 PMC2478656

[15] Zalsman G, Hawton K, Wasserman D, van Heeringen K, Arensman E, Sarchiapone M, Carli V, Höschl C, Barzilay R, Balazs J, Purebl G, Kahn JP, Sáiz PA, Lipsicas CB, Bobes J, Cozman D, Hegerl U and Zohar J. Suicide prevention strategies revisited: 10-year systematic review. Lancet Psychiatry. 2016; 3(7): 646–59. 10.1016/S2215-0366(16)30030-X 27289303

[16] Gould MS, Greenberg T, Velting DM and Shaffer D. Youth suicide risk and preventive interventions: a review of the past 10 years. J Am Acad Child Adolesc Psychiatry. 2003; 42(4): 386–405. 10.1097/01.CHI.0000046821.95464.CF 12649626

[17] Portzky G, Audenaert K and van Heeringen K. Psychosocial and psychiatric factors associated with adolescent suicide: a case-control psychological autopsy study. J Adolesc. 2009; 32(4): 849–62. 10.1016/j.adolescence.2008.10.007 19027150

[18] Tong A, Craig J, Flemming K, McInnes E and Oliver S. Enhancing transparency in reporting the synthesis of qualitative research: ENTREQ. BMC Med Res Methodol. 2012; 12(1): 181 10.1186/1471-2288-12-181 23185978 PMC3552766

[19] Moher D, Liberati A, Tetzlaff J, Altman DG and PRISMA Group. Preferred reporting items for systematic reviews and meta-analyses: the PRISMA statement. Ann Intern Med. 2009; 151(4): 264–269. 19622511 10.7326/0003-4819-151-4-200908180-00135

[20] Critical Appraisal Skills Programme. CASP for Qualitative Studies Checklist 2018 https://casp-uk.net/wp-content/uploads/2018/03/CASP-Qualitative-Checklist-2018_fillable_form.pdf

[21] Everall RD, Bostik KE and Paulson BL. I'm sick of being me: Developmental themes in a suicidal adolescent. Adolescence. 2005; 40(160): 693 16468666

[22] Jo KH, An GJ and Sohn KC. Qualitative content analysis of suicidal ideation in Korean college students. Collegian.2011; 18(2): 87–92. 10.1016/j.colegn.2010.11.001 21706996

[23] Schwartz KA, Pyle SA, Dowd MD and Sheehan K. Attitudes and beliefs of adolescents and parents regarding adolescent suicide. Pediatrics. 2010; 125(2): 221–227. 10.1542/peds.2008-2248 20064861

[24] Bostik KE and Everall RD. Healing from suicide: Adolescent perceptions of attachment relationships. British Journal of Guidance & Counselling. 2007; 35(1): 79–96. 10.1080/03069880601106815

[25] Everall RD, Bostik KE and Paulson BL. Being in the safety zone: Emotional experiences of suicidal adolescents and emerging adults. Journal of Adolescent Research. 2006; 21(4): 370–392. 10.1177/0743558406289753

[26] Fullagar S, Gilchrist H and Sullivan G. The construction of youth suicide as a community issue within urban and regional Australia. Australian e-Journal for the Advancement of Mental Health. 2007; 6(2): 107–118. http://www.auseinet.com/journal/vol6iss2/fullagar.pdf

[27] Strickland CJ and Cooper M. Getting into Trouble: Perspectives on Stress and Suicide Prevention Among Pacific Northwest Indian Youth. J Transcult Nurs.2011; 22(3): 240–247. 10.1177/1043659611404431 21519060

[28] Strickland CJ, Walsh E and Cooper M. Healing fractured families: parents' and elders' perspectives on the impact of colonization and youth suicide prevention in a pacific northwest American Indian tribe. J Transcult Nurs.2006; 17(1): 5–12. 10.1177/1043659605281982 16410431

[29] Tingey L, Cwik MF, Goklish N, Larzelere-Hinton F, Lee A, Suttle R, Walkup JT and Barlow A. Risk pathways for suicide among Native American adolescents. Qual Health Res.2014; 24(11): 1518–1526. 10.1177/1049732314548688 25168705

[30] Walls ML, Hautala D and Hurley J. “Rebuilding our community”: Hearing silenced voices on Aboriginal youth suicide. Transcult Psychiatry.2014; 51(1): 47–72. 10.1177/1363461513506458 24097414 PMC4096116

[31] Gulbas LE, Hausmann-Stabile C, De Luca SM, Tyler TR and Zayas LH. An exploratory study of nonsuicidal self-injury and suicidal behaviors in adolescent latinas. Am J Orthopsychiatry. 2015; 85(4): 302–314. 10.1037/ort0000073 26052816 PMC4501885

[32] Gulbas LE and Zayas LH. Examining the interplay among family, culture, and Latina teen suicidal behavior. Qual Health Res.2015; 25(5): 689–699. 10.1177/1049732314553598 25288407 PMC4382415

[33] Zayas L, Gulbas LE, Fedoravicius N and Cabassa LJ. Patterns of distress, precipitating events, and reflections on suicide attempts by young Latinas. Soc Sci Med. 2010; 70(11): 1773–1779. 10.1016/j.socscimed.2010.02.013 20347199 PMC2862781

[34] Herrera A, Dahlblom K, Dahlgren L and Kullgren G. Pathways to suicidal behaviour among adolescent girls in Nicaragua. Soc Sci Med. 2006; 62(4): 805–814. 10.1016/j.socscimed.2005.06.055 16098648

[35] Keyvanara M and Haghshenas A. Sociocultural contexts of attempting suicide among Iranian youth: A qualitative study. East Mediterr Health J. 2011; 17(6): 529–535. 21796972

[36] Orri M, Paduanello M, Lachal J, Falissard B, Sibeoni J and Revah-Levy A. Qualitative approach to attempted suicide by adolescents and young adults: The (neglected) role of revenge. PLoS ONE. 2014; 9(5): e96716 10.1371/journal.pone.0096716 24802777 PMC4011950

[37] Jegannathan B, Kullgren G and Dahlblom K. How do young people in Cambodia perceive the impact of societal attitudes, media and religion on suicidal behaviour? Int J Soc Psychiatry.2016; 62(2): 114–122. 10.1177/0020764015597952 26238990

[38] Holliday C and Vandermause R. Teen experiences following a suicide attempt. Arch Psychiatr Nurs. 2015; 29(3): 168–173. 10.1016/j.apnu.2015.02.001 26001716

[39] Roen K, Scourfield J and McDermott E. Making sense of suicide: A discourse analysis of young people's talk about suicidal subjecthood. Soc Sci Med. 2008; 67(12): 2089–2097. 10.1016/j.socscimed.2008.09.019 18950923

[40] Montreuil M, Butler KJD, Stachura M and Gros CP. Exploring Helpful Nursing Care in Pediatric Mental Health Settings: The Perceptions of Children with Suicide Risk Factors and Their Parents. Issues Ment Health Nurs. 2015; 36(11): 849–859. 10.3109/01612840.2015.1075235 26631856

[41] Sukhawaha S, Arunpongpaisal S and Rungreangkulkij S. Attempted Suicide Triggers in Thai Adolescent Perspectives. Arch Psychiatr Nurs. 2016; 30(3): 334–341. 10.1016/j.apnu.2015.12.005 27256938

[42] Coggan C, Patterson P and Fill J. Suicide: qualitative data from focus group interviews with youth. Soc Sci Med.1997; 45(10): 1563–1570. 10.1016/S0277-9536(97)00098-1 9351146

[43] Matel-Anderson DM and Bekhet AK. Resilience in Adolescents Who Survived a Suicide Attempt from the Perspective of Registered Nurses in Inpatient Psychiatric Facilities. Issues Ment Health Nurs. 2016; 37(11): 839–846. 10.1080/01612840.2016.1193578 27351243

[44] Shilubane HN, Ruiter RAC, Bos AER, Reddy PS and Van Den Borne B. High school students' knowledge and experience with a peer who committed or attempted suicide: A focus group study. BMC Public Health. 2014; 14(1): 10.1186/1471-2458-14-1081 25326033 PMC4216354

[45] White J and Morris J. Precarious spaces: Risk, responsibility and uncertainty in school-based suicide prevention programs. Soc Sci Med. 2010; 71(12): 2187–2194. 10.1016/j.socscimed.2010.09.046 21050629

[46] Shilubane HN, Ruiter RA, Bos AE, van den Borne B, James S and Reddy PS. Psychosocial determinants of suicide attempts among black South African adolescents: A qualitative analysis. J Youth Stud. 2012; 15(2): 177–189. 10.1080/13676261.2011.634400

[47] Bergmans Y, Langley J, Links P and Lavery JV. The perspectives of young adults on recovery from repeated suicide-related behavior. Crisis. 2009; 30(3): 120–127. 10.1027/0227-5910.30.3.120 19767267

