# Supplementary material for: A qualitative systematic review of experiences and perceptions of youth suicide
Source: PLoS One. 2019 Jun 12;14(6):e0217568. doi: 10.1371/journal.pone.0217568 (PMC6561633; doi:10.1371/journal.pone.0217568)
Supplement: S1 Table — (DOCX) [file pone.0217568.s001.docx]

| **No** | | **Item** | | **Guide and description** | | **Response (Page No. in manuscript)** |
| --- | --- | --- | --- | --- | --- | --- |
| **1** | | Aim | | State the research question the synthesis addresses. | | To review and synthesize qualitative studies that explored the experiences and perceptions of suicide in people 25 years old and younger. (abstract, p.2; introduction, p.8-9) |
| **2** | | Synthesis methodology | | Identify the synthesis methodology or theoretical framework which underpins the synthesis, and describe the rationale for choice of methodology *(e.g. meta-ethnography, thematic synthesis, critical interpretive synthesis, grounded theory synthesis, realist synthesis, meta-aggregation, meta-study, framework synthesis).* | | “Thomas and Harden”. (Methods, p.9) |
| **3** | | Approach to searching | | Indicate whether the search was pre-planned (*comprehensive search strategies to seek all available studies)* or iterative (*to seek all available concepts until they theoretical saturation is achieved)*. | | Pre-planned (Methods, p.9-10) |
| **4** | | Inclusion criteria | | Specify the inclusion/exclusion criteria *(e.g. in terms of population, language, year limits, type of publication, study type).* | | See Methods, p. 9 |
| **5** | | Data sources | | Describe the information sources used (e.g. *electronic databases (MEDLINE, EMBASE, CINAHL, psycINFO, Econlit), grey literature databases (digital thesis, policy reports), relevant organisational websites, experts, information specialists, generic web searches (Google Scholar) hand searching, reference lists)* and when the searches conducted; provide the rationale for using the data sources. | | See “Search strategy,” p.9-10 |
| **6** | | Electronic Search strategy | | Describe the literature search *(e.g. provide electronic search strategies with population terms, clinical or health topic terms, experiential or social phenomena related terms, filters for qualitative research, and search limits)*. | | Databases and Mesh terms reported, and no time parameters used (p.9-10). S2 Table - Sample search strategy provided. |
| **7** | | Study screening methods | | Describe the process of study screening and sifting *(e.g. title, abstract and full text review, number of independent reviewers who screened studies).* | | See Figure 1 and p.10 |
| **8** | | Study characteristics | | Present the characteristics of the included studies *(e.g. year of publication, country, population, number of participants, data collection, methodology, analysis, research questions).* | | This information is included in text (p.15) and Table 5 (p.16-21) |
| **9** | | Study selection results | | Identify the number of studies screened and provide reasons for study exclusion *(e,g, for comprehensive searching, provide numbers of studies screened and reasons for exclusion indicated in a figure/flowchart; for iterative searching describe reasons for study exclusion and inclusion based on modifications t the research question and/or contribution to theory development).* | | See Figure 1 and page 10 |
| **10** | | Rationale for appraisal | | Describe the rationale and approach used to appraise the included studies or selected findings *(e.g. assessment of conduct (validity and robustness), assessment of reporting (transparency), assessment of content and utility of the findings).* | | See quality appraisal (p.10) and Tables 1-4. |
| **11** | Appraisal items | | State the tools, frameworks and criteria used to appraise the studies or selected findings *(e.g. Existing tools: CASP, QARI, COREQ, Mays and Pope* [25]*; reviewer developed tools; describe the domains assessed: research team, study design, data analysis and interpretations, reporting).* | | CASP Qualitative tool <https://casp-uk.net/wp-content/uploads/2018/01/CASP-Qualitative-Checklist-2018.pdf>  See quality appraisal (p.10). | |
| **12** | Appraisal process | | Indicate whether the appraisal was conducted independently by more than one reviewer and if consensus was required. | | See quality appraisal (p.10). | |
| **13** | Appraisal results | | Present results of the quality assessment and indicate which articles, if any, were weighted/excluded based on the assessment and give the rationale. | | No articles were excluded on the basis of quality assessment alone. Figure 1, Tables 1-4 and p.10 | |
| **14** | Data extraction | | Indicate which sections of the primary studies were analysed and how were the data extracted from the primary studies? *(e.g. all text under the headings “results /conclusions” were extracted electronically and entered into a computer software).* | | Data extraction and synthesis (p.22) | |
| **15** | Software | | State the computer software used, if any. | | No software was used | |
| **16** | Number of reviewers | | Identify who was involved in coding and analysis. | | p.22 | |
| **17** | Coding | | Describe the process for coding of data *(e.g. line by line coding to search for concepts).* | | See page 22 – Thomas and Harden’s methods for thematic synthesis, the ‘Results’ section of each article was then analysed using line-by-line coding. | |
| **18** | Study comparison | | Describe how were comparisons made within and across studies *(e.g. subsequent studies were coded into pre-existing concepts, and new concepts were created when deemed necessary).* | | Upon completion of the line-by-line coding, each subtheme was re-examined, and each piece of text compared to others in that category for similarities and differences. p. 22 | |
| **19** | Derivation of themes | | Explain whether the process of deriving the themes or constructs was inductive or deductive. | | The process of deriving the themes or constructs was inductive. | |
| **20** | Quotations | | Provide quotations from the primary studies to illustrate themes/constructs, and identify whether the quotations were participant quotations of the author’s interpretation. | | See pages 24, 25, 27, 33 | |
| **21** | Synthesis output | | Present rich, compelling and useful results that go beyond a summary of the primary studies (e.g. *new interpretation, models of evidence, conceptual models, analytical framework, development of a new theory or construct).* | | See pages 24 – 30 | |
