# Supplementary material for: A qualitative systematic review of experiences and perceptions of youth suicide
Source: PLoS One. 2019 Jun 12;14(6):e0217568. doi: 10.1371/journal.pone.0217568 (PMC6561633; doi:10.1371/journal.pone.0217568)
Supplement: S2 Table — (DOCX) [file pone.0217568.s002.docx]

| ((((Suicidal Ideation [mh] OR suicide [mh] OR Suicide, Attempted [mh])) AND (risk factors [mh] OR trigger* [tiab])) AND (Adolescence [tiab] OR Adolescent [mh] OR teenager* [tiab])) AND (perception* [tiab] OR qualitative research [mh] OR qualitative [tiab] OR phenomenolog* [tiab] OR ethnograph* [tiab] OR grounded theory [mh] OR Anthropology, Cultural [mh] OR grounded theory [tiab]) |
| --- |

**Supplementary Table 2: PubMed search strategy**
